# Repeated TRPA1 Blockade Reduces Nociception and Neuroinflammation in a Relapsing–Remitting Mouse Model of Multiple Sclerosis

**DOI:** 10.3390/ijms27219592

**Published:** 2026-10-27

**Authors:** Ana Clara Machado Luiz, Diulle Spat Peres, Sabrina Qader Kudsi, Ricardo Iuri Felix Moraes, Caren Antoniazzi, Leonardo Gomes Pereira, Helen Alves Brito, Micheli Mainardi Pillat, Mariana Martins Flores, Patrícia Rodrigues, Gabriela Trevisan

**Affiliations:** 1 Graduate Program in Pharmacology, Federal University of Santa Maria (UFSM), Santa Maria 97105-900, Brazil; machado.anaclara@hotmail.com (A.C.M.L.); iuri.felix@acad.ufsm.br (R.I.F.M.);; 2 The Alan Edwards Centre for Research on Pain, Lyman Duff Medical Building, McGill University, Montréal, QC H3A 2B4, Canada; carenantoniazzi@gmail.com; 3 Graduate Program in Veterinary Medicine, Federal University of Santa Maria (UFSM), Santa Maria 97105-900, Brazil; 4 Graduate Program in Biological Sciences, Federal University of Santa Maria (UFSM), Santa Maria 97105-900, Brazil

**Keywords:** analgesia, TRP channels, A-967079, allodynia, astrocytes, pain

## Abstract

Relapsing–remitting multiple sclerosis (RRMS) is a chronic autoimmune and neurodegenerative disease characterized by neuroinflammation and demyelination. Neuropathic pain is a frequent and poorly managed symptom associated with oxidative stress. Transient receptor potential ankyrin 1 (TRPA1), which is activated by oxidative mediators, contributes to nociceptive signaling and neuroinflammatory processes in the relapsing–remitting experimental autoimmune encephalomyelitis (RR-EAE) model. This study investigated the effects of repeated pharmacological blockade of TRPA1 on nociceptive behaviors and disease-associated pathological mechanisms using an RR-EAE mouse model. Female C57BL/6 mice received the TRPA1 antagonist A-967079 (30 mg/kg, intragastric) from day 16 to day 36 post-induction. Behavioral, molecular, and biochemical analyses were performed. Repeated administration of A-967079 attenuated mechanical and cold allodynia without impairing locomotor function or altering body weight. TRPA1 antagonism modulated glial marker expression and plasma pro-inflammatory cytokine levels. A-967079 attenuated oxidative stress, reducing hydrogen peroxide, 4-hydroxynonenal, advanced oxidation protein product levels (TRPA1 endogenous agonists), and NADPH–cytochrome c reductase activity. These findings demonstrate that repeated TRPA1 antagonism effectively modulates neuropathic pain-like behaviors and targets key pathological mechanisms associated with RRMS. This approach closely reflects clinical management, as treatment was initiated after disease onset, when symptoms were already present. Our results support TRPA1 antagonism as a promising therapeutic strategy for pain management in RRMS.

## 1. Introduction

Relapsing–remitting multiple sclerosis (RRMS) is a chronic inflammatory disease of the central nervous system (CNS), characterized by demyelinating lesions distributed throughout the white matter [1,2]. RRMS is the most common form of multiple sclerosis (MS) without known pathophysiology [2]. RRMS follows a cyclic pattern of symptom exacerbation (relapses) followed by periods of partial or complete disappearance of these signs (remission) [3]. Typically, the first symptoms manifest between 20 and 40 years of age, with a higher prevalence in women than in men [4,5]. RRMS encompasses cognitive, motor, and sensory impairments, with neuropathic pain being particularly debilitating [6].

Neuropathic pain is often difficult to manage and can significantly interfere with daily activities. In patients with MS, its pharmacological management relies mainly on the use of anticonvulsants, such as gabapentin and pregabalin, and antidepressants, such as duloxetine, which are considered first-line therapies [7]. However, current therapeutic approaches have limited efficacy and are often associated with adverse effects, reducing patients’ quality of life [8,9]. Given these limitations, there remains a need for novel therapeutic strategies to improve neuropathic pain management in RRMS patients.

One promising therapeutic target for neuropathic pain is transient receptor potential ankyrin 1 (TRPA1), a non-selective ion channel activated by oxidative and endogenous compounds, including hydrogen peroxide (H_2_O_2_), advanced oxidation protein products (AOPPs), and 4-hydroxynonenal (4-HNE) [10,11,12,13,14]. TRPA1 is expressed in neuronal and non-neuronal cells [15,16], including astrocytes [17] and oligodendrocytes [18], and is implicated in neuroinflammation [19]. Consistent with these findings, TRPA1 knockout mice (*Trpa1*^−/−^) subjected to the relapsing–remitting experimental autoimmune encephalomyelitis (RR-EAE) model do not develop allodynia (mechanical and cold) or neuroinflammation [20].

Moreover, acute treatment with the TRPA1 antagonist A-967079 reduced nociception, neuroinflammation, and anxiety- and depressive-like behaviors in the RR-EAE model [21,22]. However, previous studies have focused only on acute pharmacological blockade of TRPA1 or genetic deletion, leaving the effects of repeated TRPA1 antagonism on long-term neuropathic-like symptoms and neuroinflammation unexplored. Therefore, this study aimed to investigate the effects of repeated administration of the selective TRPA1 antagonist A-967079 on neuropathic-like symptoms induced by the RR-EAE. By addressing this gap, this study may contribute to understanding the therapeutic potential of repeated TRPA1 antagonism for managing neuropathic pain associated with RRMS.

## 2. Results

### 2.1. Repeated Administration of the TRPA1 Antagonist A-967079 Induced an Antinociceptive Effect in the RR-EAE Model

**Figure 1:**
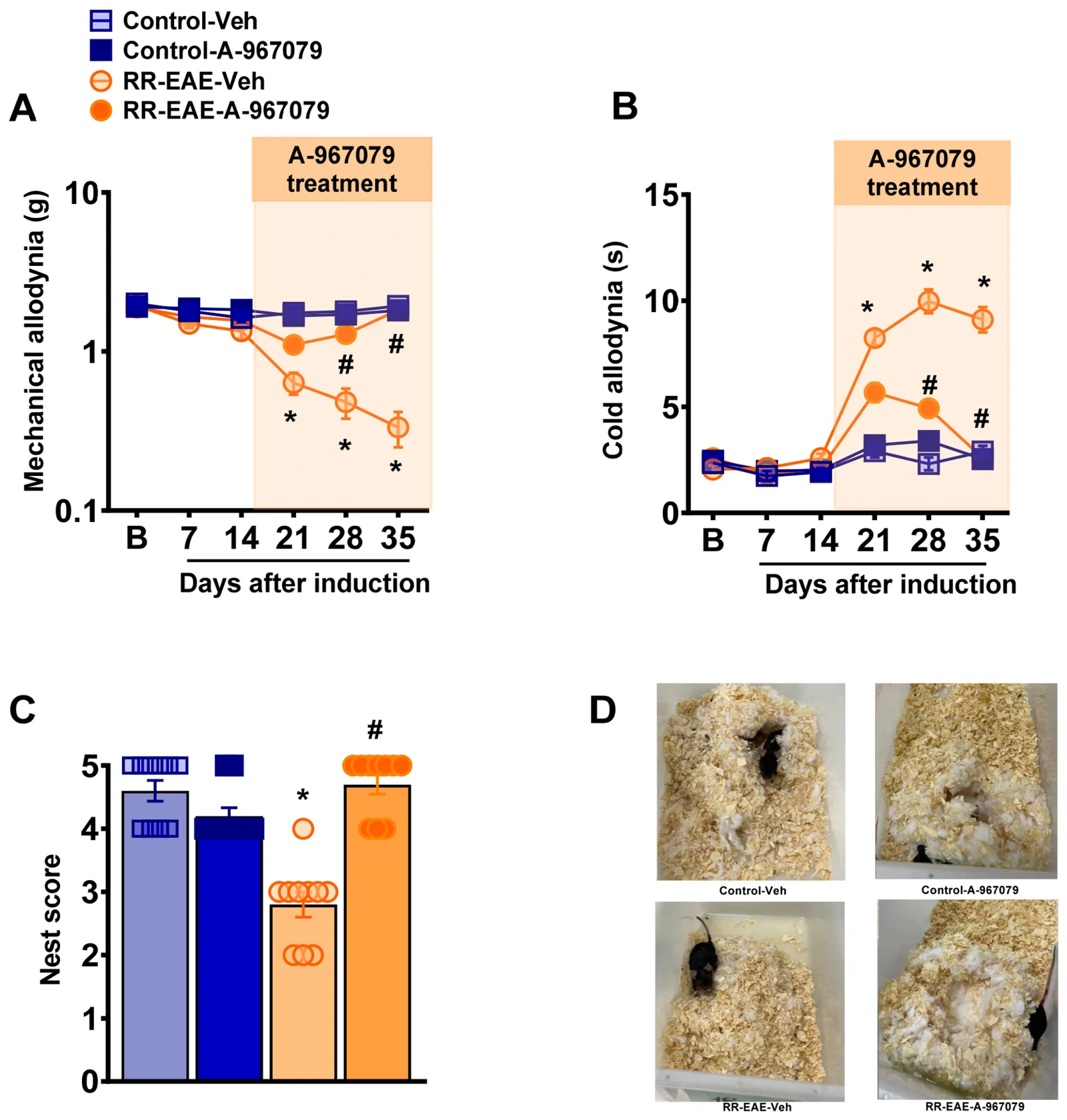
Repeated administration of A-967079 alleviates nociceptive hypersensitivity and improves nest-building behavior in RR-EAE mice. (**A**) Mechanical allodynia was assessed using von Frey filaments (grams, g). Vehicle-treated RR-EAE mice showed decreased withdrawal thresholds on days 21, 28, and 35 post-induction, indicating increased sensitivity to mechanical stimuli. A-967079 treatment significantly attenuated this response. (**B**) Cold allodynia was evaluated using the acetone test (seconds, s). RR-EAE mice showed increased response times, which A-967079 treatment significantly reduced. (**C**) Nest-building behavior was impaired in RR-EAE mice, as evidenced by lower nest scores. A-967079 significantly improved nest-building performance. (**D**) Representative images of the nests built by each group on day 34 post-induction. Baseline measurements ((**B**) in the graph) were performed before induction. Data are expressed as mean ± SEM (*n* = 10). * *p* < 0.05 indicates differences when comparing Control-Veh with RR-EAE-Veh. ^#^ *p* < 0.05 indicates differences when comparing RR-EAE-Veh with RR-EAE-A-967079. A mixed-effects model followed by Bonferroni’s multiple comparisons test was used for graphs (**A**,**B**). The Kruskal–Wallis test followed by Dunn’s multiple comparisons test was used for graph (**C**).

Repeated administration of the TRPA1 antagonist A-967079 produced a significant antinociceptive effect in RR-EAE mice. As shown in Figure 1A, RR-EAE-Veh mice exhibited mechanical allodynia from day 21 to day 35 post-induction, indicating increased mechanical sensitivity compared to the Control-Veh group (day 21: *p* = 0.0043, 95% CI: 0.3015 to 1.923; day 28: *p* = 0.0008, 95% CI: 0.5334 to 2.070; day 35: *p* < 0.0001, 95% CI: 1.035 to 2.166). In contrast, treatment with A-967079 significantly reduced mechanical allodynia compared with the RR-EAE-Veh group on day 28 (*p* = 0.0054, 95% CI: −1.410 to −0.2011) and day 35 post-induction (*p* = 0.0002, 95% CI: −2.211 to −0.7574) (Figure 1A). Similarly, RR-EAE-Veh mice exhibited increased cold sensitivity compared with the Control-Veh group at all evaluated time points (day 21: *p* < 0.0001, 95% CI: −7.448 to −3.224; day 28: *p* < 0.0001, 95% CI: −11.14 to −4.183; day 35: *p* = 0.0004, 95% CI: −9.576 to −2.860) (Figure 1B). Treatment with A-967079 significantly reduced cold allodynia compared with the RR-EAE-Veh group on days 28 (*p* = 0.0015, 95% CI: 1.808 to 8.272) and 35 post-induction (*p* = 0.0015, 95% CI: 2.329 to 10.72) (Figure 1B). In addition, RR-EAE mice exhibited impaired nest-building behavior, as reflected by significantly lower nest scores than those of the Control-Veh group (*p* < 0.0001) (Figure 1C). Treatment with A-967079 significantly improved this behavior compared with the RR-EAE-Veh group (*p* < 0.0001, restoring nest scores to levels comparable to those of the control animals (Figure 1C). Representative images of nest-building behavior in animals from each group are shown in Figure 1D.

### 2.2. The Open Field Test Revealed Changes in the Behavioral Activity of RR-EAE Animals, Which Was Improved by TRPA1 Antagonism

**Figure 2:**
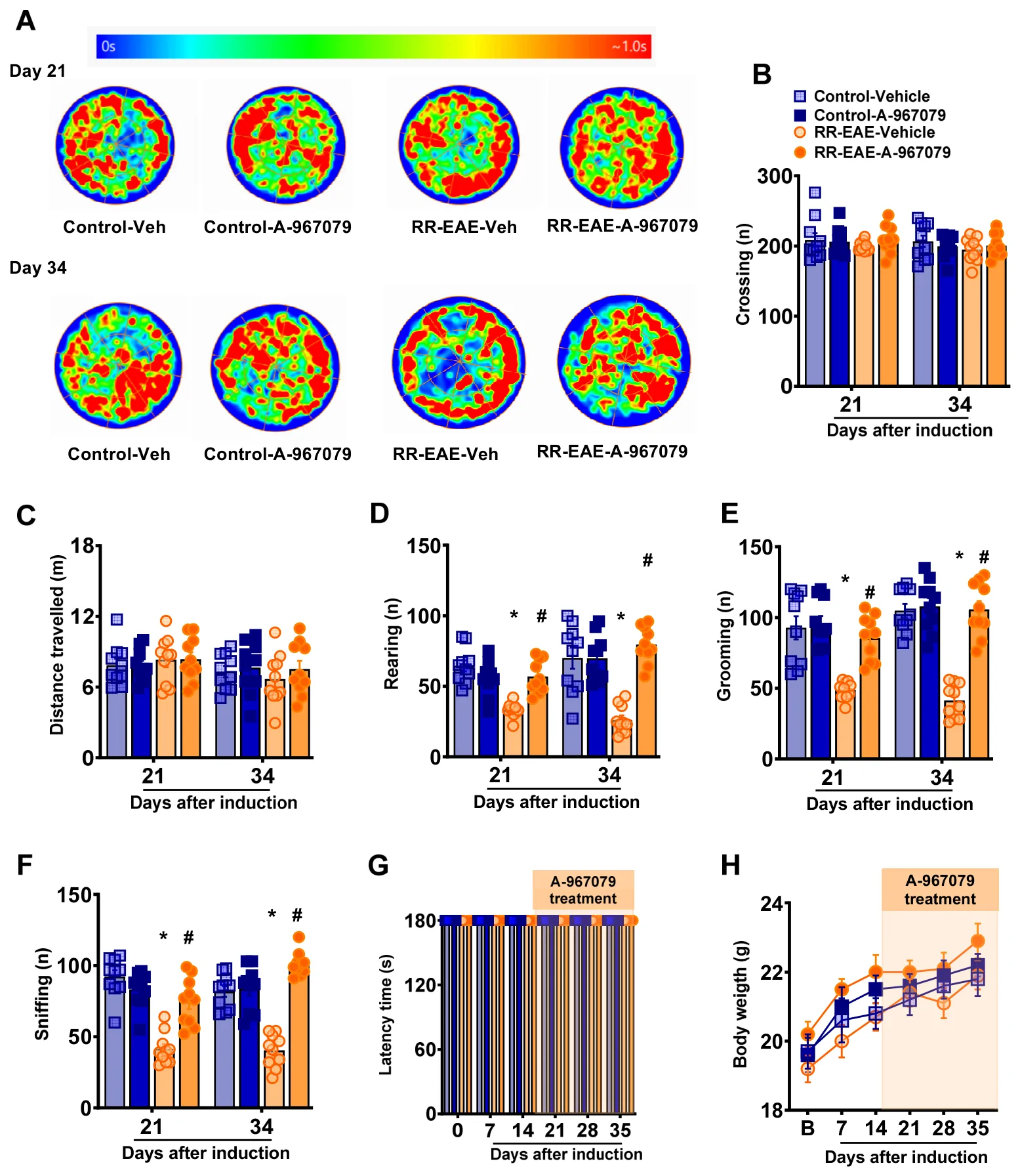
Evaluation of locomotor activity and spontaneous behaviors in the open field test and motor performance in the rotarod test. (**A**) Representative heatmaps of locomotor activity on days 21 and 34 post-induction. (**B**) Number of crossings (*n*) and (**C**) total distance traveled (meters, m) showed no significant differences among groups at either time point. (**D**–**F**) Vehicle-treated RR-EAE animals exhibited a significant reduction in (**D**) rearing (number, *n*), (**E**) grooming (*n*), and (**F**) sniffing (*n*) behaviors on days 21 and 34 p.i. compared with control animals. Treatment with the TRPA1 antagonist A-967079 partially improved these behaviors, with significant increases in rearing, grooming, and sniffing on both days compared to vehicle-treated RR-EAE animals. (**G**) Latency time (seconds, s) in the rotarod test throughout the experimental protocol. Baseline measurements (**B** in the graph) were made before the induction. (**H**) Body weight (grams, g) throughout the experimental protocol. No significant differences were observed among groups in either motor performance or body weight. Data are presented as mean ± SEM (*n* = 10). * *p* < 0.05 indicates differences when comparing Control-Veh with RR-EAE-Veh. # *p* < 0.05 indicates differences when comparing RR-EAE-Veh with RR-EAE-A-967079. Two-way ANOVA followed by Bonferroni’s post hoc test (graphs (**B**–**F**)). Mixed-effects model followed by Bonferroni’s multiple comparisons test (graphs (**G**,**H**)).

Representative heatmaps of locomotor activity on days 21 and 34 post-induction are shown in Figure 2A. No significant differences were observed between the groups in the number of crossings (Figure 2B) or in the total distance traveled (Figure 2C) at any of the evaluated time points. However, RR-EAE-Veh animals showed a significant reduction in rearing behavior (day 21: *p* = 0.0003; 95% CI: 15.41 to 47.19; day 34: *p* = 0.0348, 95% CI: 2.384 to 85.22) (Figure 2D), grooming (day 21: *p* = 0.0238, 95% CI: 4.691 to 83.11; day 34: *p* < 0.0001, 95% CI: 36.86 to 90.14) (Figure 2E), and sniffing (day 21: *p* = 0.0001, 95% CI: 27.71 to 73.69; day 34: *p* = 0.0009, 95% CI: 17.49 to 65.11) (Figure 2F) compared with control groups on days 21 and 34 post-induction. Treatment with the TRPA1 antagonist A-967079 improved these behaviors, with significant increases in rearing (day 21: *p* = 0.0133, 95% CI: −43.15 to −4.246; day 34: *p* = 0.0002, 95% CI: −79.09 to −27.51), grooming (day 21: *p* = 0.0077, 95% CI: −64.80 to −8.800; day 34: *p* = 0.0003, 95% CI: −96.45 to −32.35) and sniffing (day 21: *p* = 0.0218, 95% CI: −62.48 to −3.923; day 34: *p* < 0.0001, 95% CI: −77.22 to −43.78), on both days, compared to RR-EAE-Veh animals (Figure 2D–F). No significant differences in motor performance in the rotarod test (Figure 2G) or body weight (Figure 2H) were detected among the groups throughout the experimental protocol. These findings further corroborate the open field results (Figure 2B,C), suggesting that RR-EAE induction and repeated A-967079 treatment did not significantly affect these measures of locomotor activity under these experimental conditions.

### 2.3. Repeated Treatment with the A-967079 Antagonist Significantly Modulated the Neuroinflammation and Oxidative Stress Alterations Induced by the RR-EAE Model

**Figure 3:**
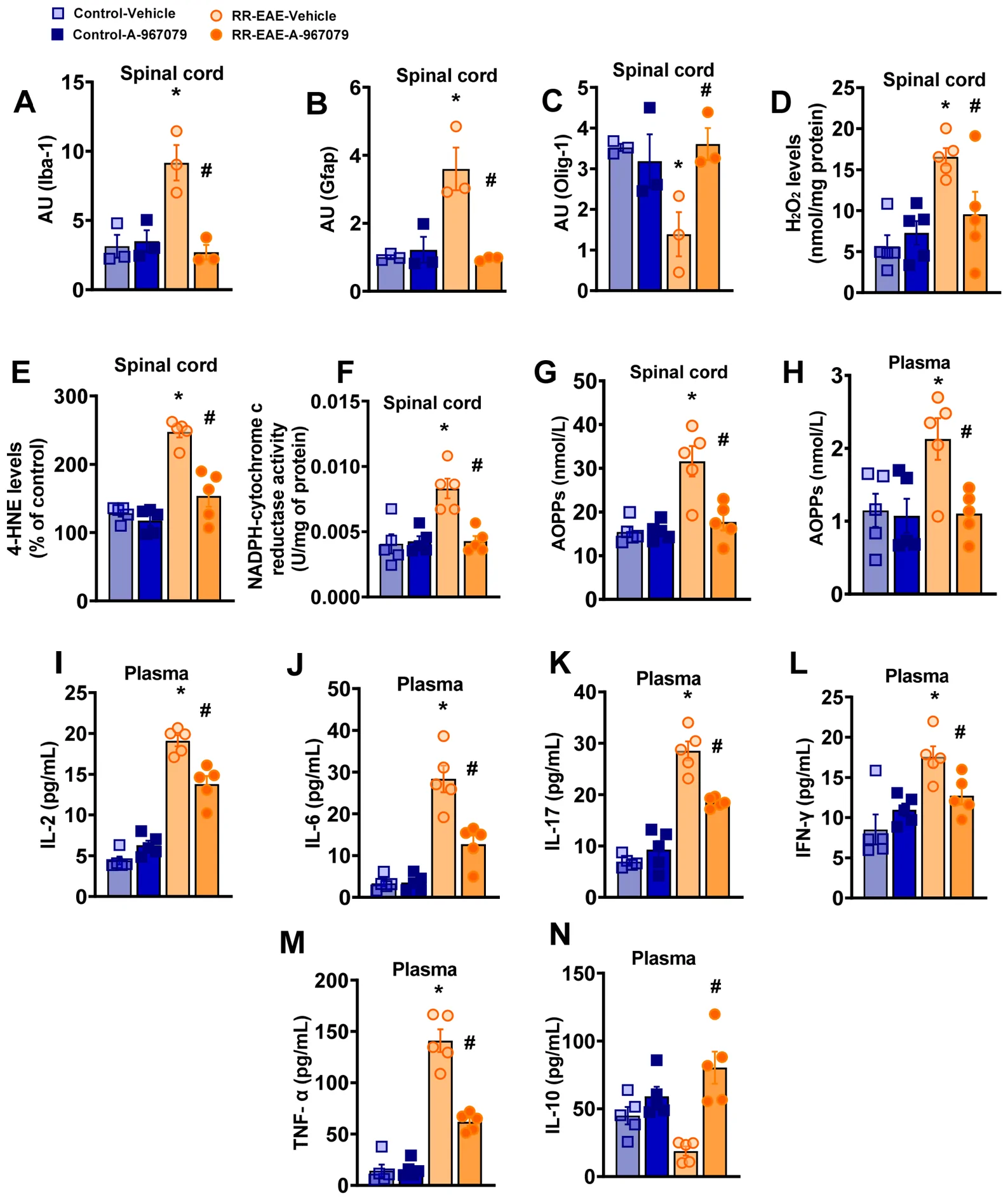
Effects of repeated A-967079 treatment on neuroinflammatory, oxidative stress, and myelin-related markers in the RR-EAE model. (**A**) mRNA expression levels of *Iba-1* (Ionized Calcium-Binding Adapter Molecule 1, expressed in arbitrary units, AU *n* = 3); (**B**) mRNA expression levels of *Gfap* (Glial Fibrillary Acidic Protein, expressed in arbitrary units, AU *n* = 3); (**C**) mRNA expression levels of *Olig-1* (Oligodendrocyte Transcription Factor 1, expressed in arbitrary units, AU *n* = 3); (**D**) hydrogen peroxide levels (H_2_O_2_, *n* = 5); (**E**) levels of 4-hydroxynonenal (4-HNE, *n* = 5); (**F**) NADPH–cytochrome c reductase activity (*n* = 5); (**G**) spinal cord levels of AOPP (*n* = 5); (**H**) plasma levels of AOPP (*n* = 5); (**I**) plasma levels of interleukin-2 (IL-2, *n* = 5); (**J**) interleukin-6 (IL-6, *n* = 5); (**K**) interleukin-17 (IL-17, *n* = 5); (**L**) interferon-gamma (IFN-γ, *n* = 5); (**M**) tumor necrosis factor-alpha (TNF-α, *n* = 5); (**N**) interleukin-10 (IL-10, *n* = 5). Data are expressed as mean ± SEM. * *p* < 0.05 indicates differences when comparing Control-Veh with RR-EAE-Veh. ^#^ *p* < 0.05 indicates differences when comparing RR-EAE-Veh with RR-EAE-A-967079. One-way ANOVA followed by Dunnett’s multiple comparisons test.

A significant increase in the *Iba-1* mRNA expression was observed in the RR-EAE group compared with the control group (*p* = 0.0029, 95% CI: −9.527 to −2.533). Treatment with A-967079 significantly reduced *Iba-1* mRNA expression compared with the RR-EAE-Veh group (*p* = 0.0019, 95% CI: 2.957 to 9.950) (Figure 3A). Similarly, *Gfap* mRNA expression was significantly increased in the RR-EAE-Veh group compared with the Control-Veh group (*p* = 0.0021, 95% CI: −4.027 to −1.166), whereas treatment with A-967079 significantly reduced its expression (*p* = 0.0019, 95% CI: 1.210 to 4.070) (Figure 3B). In addition, *Olig-1* mRNA expression was significantly reduced in the RR-EAE-Veh group compared with the Control-Veh group (*p* = 0.0251, 95% CI: 0.3012 to 3.972). Treatment with A-967079 significantly increased *Olig-1* mRNA expression compared with the RR-EAE-Veh group (*p* = 0.0208, 95% CI: −4.055 to −0.3845) (Figure 3C). In addition, the RR-EAE-Veh group exhibited significantly increased H_2_O_2_ levels in spinal cord samples compared with the Control-Veh group (*p* = 0.0010, 95% CI: −17.14 to −4.708) (Figure 3D). Treatment with A-967079 significantly reduced spinal cord H_2_O_2_ levels compared with the RR-EAE-Veh group (*p* = 0.0260, 95% CI: 0.8075 to 13.24) (Figure 3D). Similarly, 4-HNE levels in spinal cord samples were significantly increased in the RR-EAE-Veh group compared with the Control-Veh group (*p* < 0.0001, 95% CI: −154.1 to −83.89) (Figure 3E). Treatment with A-967079 significantly reduced 4-HNE levels compared with the RR-EAE-Veh group (*p* < 0.0001, 95% CI: 58.41 to 128.6) (Figure 3E). NADPH–cytochrome c reductase activity was significantly increased in the RR-EAE-Veh group compared with the Control-Veh group (*p* = 0.0003, 95% CI: −0.006324 to −0.002139) (Figure 3F). Treatment with A-967079 significantly reduced this activity compared with the RR-EAE-Veh group (*p* = 0.0004, 95% CI: 0.001934 to 0.006119) (Figure 3F). The RR-EAE-Veh group exhibited significantly increased AOPP levels in the spinal cord compared with the Control-Veh group (*p* = 0.0001, 95% CI: −23.59 to −8.708). Treatment with A-967079 significantly reduced spinal cord AOPP levels compared with the RR-EAE-Veh group (*p* = 0.0006, 95% CI: 6.417 to 21.30) (Figure 3G). Similarly, plasma AOPP levels were significantly increased in RR-EAE-Veh mice compared with the Control-Veh group (*p* = 0.0154, 95% CI: −1.778 to −0.1844). Treatment with A-967079 significantly reduced plasma AOPP levels compared with the RR-EAE-Veh group (*p* = 0.0117, 95% CI: 0.2270 to 1.821) (Figure 3H). Regarding the cytokine profile, induction of RR-EAE significantly increased the plasma levels of the pro-inflammatory cytokines IL-2 (*p* < 0.0001, 95% CI: −17.18 to −12.19), IL-6 (*p* < 0.0001, 95% CI: −31.97 to −18.03), IL-17 (*p* < 0.0001, 95% CI: −26.12 to −17.09), IFN-γ (*p* = 0.0003, 95% CI: −13.65 to −4.450), and TNF-α (*p* < 0.0001, 95% CI: −150.6 to −102.8) compared with the Control-Veh group (Figure 3I–M), concomitant with a reduction in the anti-inflammatory cytokine IL-10 (*p* = 0.0613, 95% CI: −1.128 to 53.28) (Figure 3N). In contrast, treatment with A-967079 significantly reduced pro-inflammatory cytokine levels compared with the RR-EAE-Veh group, including IL-2 (*p* = 0.0001, 95% CI: 2.835 to 7.823), IL-6 (*p* < 0.0001, 95% CI: 8.663 to 22.61), IL-17 (*p* < 0.0001, 95% CI: 5.494 to 14.52), IFN-γ (*p* = 0.0380, 95% CI: 0.2519 to 9.449), and TNF-α (*p* < 0.0001, 95% CI: 55.16 to 102.9). In addition, A-967079 treatment significantly increased IL-10 levels (*p* < 0.0001, 95% CI: −88.74 to −34.33) (Figure 3I–N).

### 2.4. Principal Component Analysis (PCA) of the Effects of Repeated Antagonist Treatment in the RR-EAE Model

After PCA of behavioral parameters, biochemical markers, and oxidative stress markers following A-967079 treatment, we identified 17 principal components, of which only PC1 and PC2 were statistically significant (Table 1). We retained two of the 17 calculated PCs, which cumulatively explained 80.45% of the total variance. PC1 accounted for 71.75% of the variance, whereas PC2 explained an additional 8.71% (Table 1).

The first principal component (PC1) exhibited a positive association with cold responses (r = 0.945), plasma TNF-α (r = 0.927), spinal cord 4-HNE (r = 0.925), plasma IL-6 (r = 0.891), plasma IL-17 (r = 0.889), spinal cord AOPP (r = 0.861), plasma IL-2 (r = 0.861), and spinal cord NADPH–cytochrome c reductase activity (r = 0.836). In contrast, von Frey responses (r = −0.970), grooming (r = −0.898), rearing (r = −0.898), and sniffing (r = −0.843) showed negative correlations with PC1. These results indicate that PC1 captures a multivariate axis, linking cold-evoked responses with oxidative stress-related and pro-inflammatory markers.

PC2 showed the strongest positive correlation with plasma IL-10 (r = 0.698), followed by nest-building behavior (r = 0.460) and plasma IL-2 (r = 0.432). These findings indicate that PC2 captures an additional pattern of variation characterized primarily by IL-10, although other variables also contributed to this component. However, we considered the PCA exploratory, given the limited sample size relative to the number of variables. Accordingly, the component structure was interpreted as hypothesis-generating rather than confirmatory.

**Figure 4:**
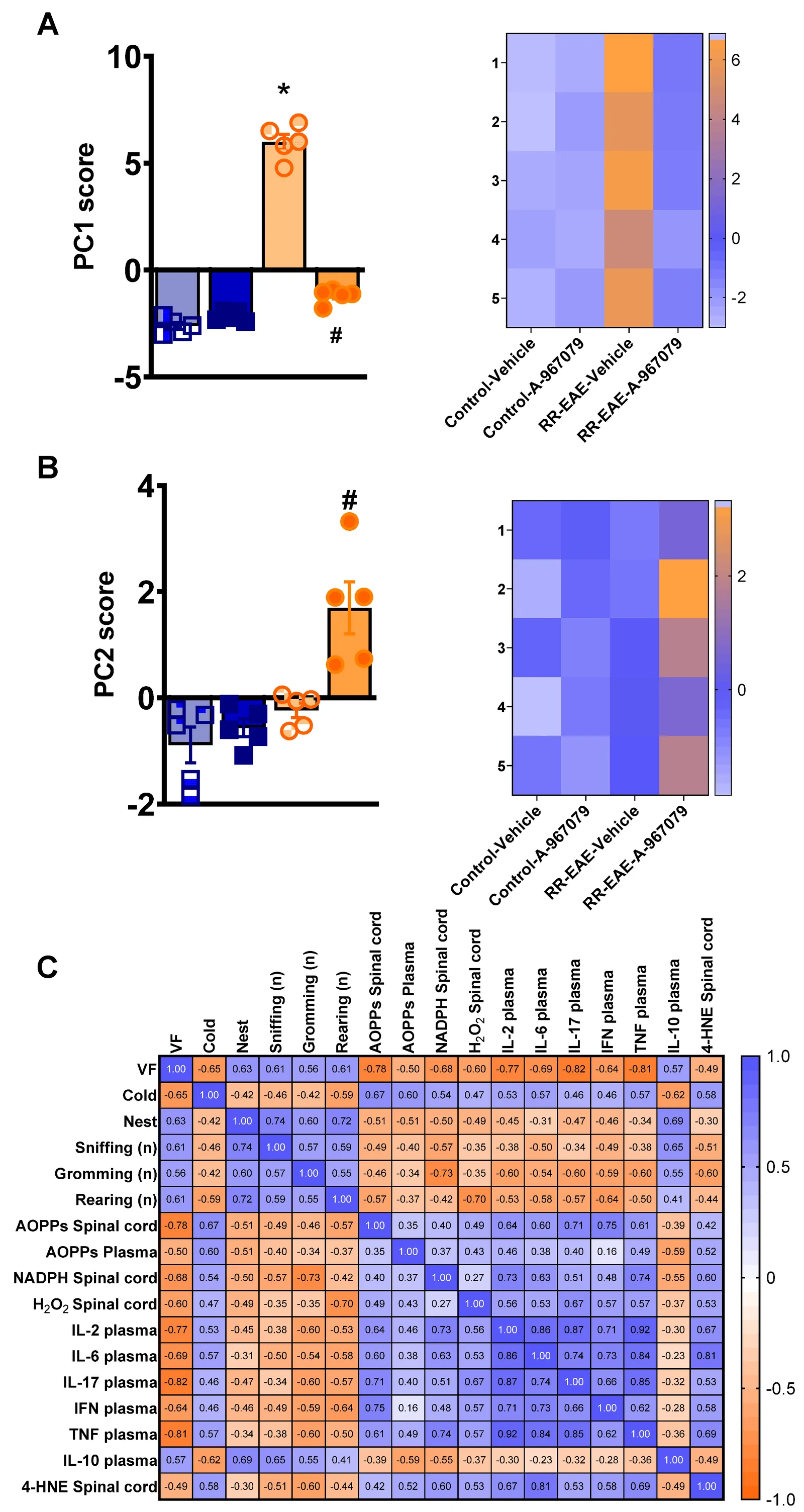
Effects of repeated A-967079 treatment in mice with relapsing–remitting experimental autoimmune encephalomyelitis (RR-EAE). (**A**) Principal Component 1 (PC1) scores and corresponding heatmap representation of individual results. (**B**) PC2 scores and corresponding heat map representation of individual results. (**C**) Heat map displaying Spearman correlation coefficients among behavioral parameters, biochemical markers, and oxidative stress indicators evaluated after repeated A-967079 treatment in the RR-EAE model. Data were analyzed using one-way ANOVA or Kruskal–Wallis test and are expressed as mean ± SEM (*n* = 5). * *p* < 0.05 indicates differences when comparing Control-Veh with RR-EAE-Veh. ^#^ *p* < 0.05 indicates differences when comparing RR-EAE-Veh with RR-EAE-A-967079.

The correlation matrix showed positive associations between the two factors. Among the cytokines, plasma IL-2 levels were positively correlated with IL-6, IL-17, and TNF-α. In addition, plasma IL-6 levels were associated with IL-2, TNF-α, and 4-HNE levels in the spinal cord. IL-17 levels also positively correlated with IL-2 and TNF-α. Thus, these results indicate an association between inflammatory and oxidative alterations in the CNS (Figure 4C).

## 3. Discussion

Chronic pain is one of the most debilitating clinical manifestations of RRMS, affecting up to 63% of patients [6]. Patients with RRMS may experience heightened sensitivity to non-noxious stimuli, manifesting as mechanical and cold allodynia, which is closely associated with activation of redox-sensitive ion channels, including TRPA1 [23]. Despite the availability of pharmacological treatments for neuropathic pain, their efficacy remains limited, and adverse effects are common, highlighting the need for safer and more effective therapeutic strategies [24,25]. In this context, we investigated the effects of repeated pharmacological blockade of TRPA1 on nociceptive responses, neuroinflammation, and oxidative pathways in the RR-EAE model.

The EAE model is the most widely used animal model for studying MS, as it reproduces key immunological and histopathological features of the disease [26]. Unlike toxin-based models, which induce demyelination without directly modeling its immune-mediated involvement, EAE enables the investigation of immune-mediated mechanisms throughout different stages of disease progression [27]. In the present study, we used the optimized RR-EAE model described by Khan et al. [28], in which immunization with MOG_35–55_ combined with the Quil A adjuvant induces central neuropathic-like pain without motor impairment. This is an important advantage over the classical CFA-based model, which is associated with pronounced motor deficits that may interfere with behavioral assessments of nociception [28,29]. The clinical score of the RR-EAE model has been characterized in previous studies, which reported changes in clinical scores over the course of disease progression [13,21,22,30]. In addition, a previous study by our group using *Trpa1*^−/−^ mice found no significant differences in clinical disease scores compared with control animals [20]. These findings provide context for interpreting the effects observed in the present study, showing a reduction in nociception.

Regarding nociception, repeated A-967079 administration produced robust antinociceptive effects, as evidenced by a significant reduction in mechanical and cold allodynia from day 21 post-immunization onward. These results align with previous studies showing the acute antinociceptive effects of TRPA1 antagonists and antioxidant compounds in the RR-EAE model [21]. Further support for TRPA1’s involvement in neuropathic-like pain comes from studies showing that mice with genetic deletion of TRPA1 do not develop allodynia after RR-EAE induction, reinforcing TRPA1’s central role in neuropathic-like pain [20]. Building on these findings, the present study shows that repeated pharmacological blockade of TRPA1 maintains antinociceptive efficacy throughout disease progression. In addition to reducing evoked nociceptive responses, A-967079 treatment improved nest-building behavior in RR-EAE mice, suggesting a beneficial effect on spontaneous, pain-related behavior. Nest-building is a relevant measure of spontaneous behavior and animal welfare in laboratory rodents and can complement conventional evoked nociceptive tests [13,31]. These findings are also consistent with previous reports of impaired nest-building behavior in the RR-EAE model [13,30].

In our work, the absence of motor impairment was further confirmed in the open field and rotarod tests. In the open field test, no changes were observed in the number of crossings or the total distance traveled, and no significant differences in motor performance were detected in the rotarod test. However, ethological behaviors such as rearing, grooming, and sniffing were reduced in RR-EAE mice, suggesting alterations in exploratory and spontaneous behavioral activity. Previous studies using RR-EAE and PMS-EAE models reported similar results, in which ethological alterations occurred independently of motor deficits [13,22,32]. Notably, repeated treatment with the TRPA1 antagonist A-967079 partially reversed the reductions in rearing, grooming, and sniffing on days 21 and 34 post-immunization without affecting locomotor activity. These findings suggest that pharmacological modulation of TRPA1 may influence not only nociception but also spontaneous behavioral changes associated with the disease.

Beyond behavioral effects, TRPA1 blockade attenuated molecular and cellular alterations associated with RRMS pathophysiology. Treatment with A-967079 reduced glial marker expression, as evidenced by decreased *Gfap* and *Iba-1*, classical markers of astrocytic and microglial reactivity, respectively. These results align with previous studies showing increased expression of these markers in the spinal cord in RR-EAE and PMS-EAE models and reduced expression after TRPA1 blockade [13,20,21,28]. Together, these findings reinforce TRPA1’s role in modulating central neuroinflammation associated with MS. At the molecular level, RR-EAE mice showed reduced *Olig-1* gene expression, consistent with alterations in oligodendrocyte-related processes associated with demyelination, as previously described [21,28]. Importantly, A-967079 treatment partially restored *Olig-1* expression, suggesting a potential modulatory effect on oligodendrocyte-related processes in the spinal cord. However, this finding does not directly demonstrate oligodendrocyte recovery and remyelination.

Furthermore, our findings support oxidative stress involvement in RR-EAE pathophysiology, as evidenced by increased NADPH–cytochrome c reductase activity and elevated H_2_O_2_ and 4-HNE levels. These oxidative mediators can activate TRPA1 and have been associated with nociceptive signaling in different experimental models [21,33,34,35]. NADPH–cytochrome c reductase is also an important enzymatic source of reactive oxygen species and plays a critical role in chronic neurodegenerative diseases, including MS [36]. In RR-EAE mice, increased activity of this enzyme has been reported in both the brain and spinal cord [21,36]. Consistent with these findings, our results showed elevated H_2_O_2_ and 4-HNE levels, along with increased NADPH–cytochrome c reductase activity in spinal cord samples. Importantly, treatment with the selective TRPA1 antagonist significantly attenuated NADPH–cytochrome c reductase activity and the accumulation of oxidative markers. These findings reinforce the role of oxidative stress in RR-EAE pathophysiology and suggest that TRPA1 blockade may disrupt redox-dependent nociceptive mechanisms, offering a potential therapeutic strategy for RRMS-associated pain.

In addition to H_2_O_2_ and 4-HNE, AOPPs are recognized as important markers and mediators of oxidative stress in both PMS-EAE and RR-EAE models [13,37]. AOPPs participate in reactive oxygen species-mediated oxidative processes and are strongly associated with neuroinflammation, disease severity, and nociceptive behavior. In the PMS-EAE model, AOPP levels are significantly increased and positively correlated with worsening clinical scores, reduced paw strength, and increased mechanical and cold allodynia, consistent with a neuropathic-like pain phenotype [38]. Complementary studies in the RR-EAE model show that inhibiting NADPH–cytochrome c reductase activity or directly neutralizing AOPPs significantly reduces their levels, along with improvements in behavioral deficits, neuroinflammation, and demyelination [13]. Consistent with these observations, the present study shows that prolonged pharmacological blockade of TRPA1 reduced AOPP levels in both the spinal cord and plasma of RR-EAE mice. Our results suggest a functional interaction between TRPA1 activation and NADPH–cytochrome c reductase activity-mediated oxidative mechanisms in the pathophysiology of the RR-EAE model.

Mechanistically, TRPA1 blockade likely attenuates oxidative and inflammatory markers by preventing sustained Ca^2+^ influx, a key signaling event downstream of TRPA1 activation [12,35]. Intracellular Ca^2++^ elevation activates multiple Ca^2+^-dependent enzymes, including NADPH–cytochrome c reductase, phospholipases, and kinases, which collectively amplify oxidative stress and tissue damage [10,39]. In parallel, Ca^2+^ signaling centrally activates transcriptional regulatory pathways, such as NF-κB, which drives expression of pro-inflammatory cytokines and redox-related enzymes, sustaining a feed-forward cycle of neuroinflammation and oxidative stress [40,41,42]. By inhibiting TRPA1, pharmacological blockade may modulate this Ca^2+^-dependent signaling network, limiting the enzymatic generation of reactive species and transcriptional upregulation of inflammatory mediators [15,20,21]. This proposed mechanism may help explain the reduced lev_22_s of H_2_O_2_, 4-HNE, AOPP, and inflammatory mediators observed in the present study. However, because we did not directly assess Ca^2+^ signaling, NADPH–cytochrome c reductase activity, or NF-κB signaling in our experiments, further studies are needed to determine whether these pathways mediate the effects of TRPA1 blockade in RR-EAE–associated neuropathic-like pain.

In this model, immunization with MOG_35–55_ combined with Quil A adjuvant increases blood–brain barrier permeability due to T-cell infiltration into the CNS [28]. In the present study, treatment with the TRPA1 antagonist A-967079 significantly reduced pro-inflammatory cytokine levels, including IL-2, IL-6, IL-17, IFN-γ, and TNF-α. Additionally, increased levels of the anti-inflammatory cytokine IL-10 were observed following antagonist treatment. IL-10 plays a central role in regulating T helper (Th1 and Th2) cell responses, which release inflammatory mediators such as cytokines and chemokines that contribute to blood–brain barrier disruption [42].

Despite these consistent results, some limitations should be considered. The sample size in the RT-qPCR experiments was small, with only three animals per group, whereas five animals per group were used in the biochemical and cytokine analyses. The RT-qPCR sample size was based on previously published studies using the same experimental model (RR-EAE) [22,30], and we did not perform an a *priori* power calculation, which may limit the detection of biological differences and increase the influence of interindividual variability. The present study evaluated only one TRPA1 antagonist, and future studies using additional antagonists will strengthen evidence for pharmacological specificity. Furthermore, only one dose of A-967079 (30 mg/kg) was evaluated, and no dose–response assessment was performed. Future studies evaluating multiple doses and additional pharmacological antagonists or genetic approaches are required to characterize the dose-dependent effects further and strengthen the evidence for the specificity of TRPA1 blockade. Moreover, behavioral and molecular assessments were conducted at specific time points, limiting the evaluation of the temporal dynamics of TRPA1 blockade during disease progression.

Furthermore, pharmacokinetic and pharmacodynamic parameters were not assessed during repeated A-967079 administration. Plasma and CNS concentrations of the drug, unbound drug exposure, and TRPA1 receptor occupancy or target engagement were not determined. Therefore, the relationship between drug exposure and the observed antinociceptive effects, as well as the relative contribution of peripheral and central TRPA1 blockade, remains unclear. In addition, potential drug accumulation, tolerance development, and changes in pharmacodynamic responsiveness during the 21-day treatment period were not investigated. Although blood–brain barrier permeability may change during EAE progression, its influence on A-967079 distribution in the present model remains unknown. Future studies assessing drug exposure, target engagement, tolerance of development, and blood–brain barrier integrity are warranted to clarify these aspects.

Additionally, the present study did not include immunohistochemical or histological analyses to assess glial reactivity or myelin integrity directly. In this context, Gritsch et al. [43] demonstrated that oligodendrocyte ablation can induce central nociceptive hypersensitivity independently of functional innate or adaptive immune responses, highlighting a potential link between oligodendrocyte dysfunction and nociceptive alterations. Our research group previously performed immunohistochemical and histological analyses of glial and myelin-related changes in knockout (*Trpa1*^−/−^) and wild-type (*Trpa1*^+/+^) animals [11,14], but we did not conduct these assessments in the present study following A-967079 treatment. However, the observed changes in *Iba-1*, *Gfap*, and *Olig-1* mRNA expression can be interpreted as changes in gene expression. Similarly, the increase in IL-10 following A-967079 treatment does not demonstrate myelin restoration. Additional histological and molecular assessments are needed to determine whether TRPA1 pharmacological blockade affects myelin integrity or remyelination.

Furthermore, the mechanisms underlying the effects of TRPA1 blockade remain to be elucidated, as Ca^2+^ signaling, NADPH–cytochrome c reductase activation, and NF-κB signaling were not directly assessed in the present study. Additionally, growing evidence indicates that nociception exhibits sex-dependent differences, which were not addressed in this study but may influence inflammatory and nociceptive responses. Therefore, future investigations exploring different temporal windows, multiple pharmacological approaches, and the influence of sex-dependent differences, as well as complementary mechanistic and histological assessments, will be important further to elucidate the role of TRPA1 in MS-associated pain and validate these findings before clinical translation can be considered.

Taken together, these findings support TRPA1’s role in nociceptive hypersensitivity and neuroinflammatory alterations in the RR-EAE model. TRPA1 antagonism produced antinociceptive effects without detectable motor impairment and attenuated molecular and biochemical markers associated with inflammation and oxidative stress. Thus, TRPA1 may be a promising therapeutic target for MS-associated pain. Further studies are needed to confirm these findings and clarify the mechanisms underlying the TRPA1 blockade in MS-associated pain.

## 4. Materials and Methods

### 4.1. Animals

Female C57BL/6 mice (20–30 g, 6–8 weeks old) were used in this study based on previous studies employing the RR-EAE model, which used female animals [13,22,28,30]. This choice was further informed by the epidemiological characteristics of MS, particularly RRMS, which accounts for approximately 80% of MS cases and is more prevalent in women, with a female-to-male ratio of approximately 2–3:1 [4]. Nevertheless, the exclusive use of female mice limits the present study and may restrict the generalizability of the findings. Animals were housed in groups of 5–10 per cage, with wood shavings as bedding. Animals were maintained at a controlled temperature (22 ± 2 °C) and on a 12-h light/dark cycle (lights on from 7:00 a.m. to 7:00 p.m.), with ad libitum access to standard laboratory chow (Puro Lab 22 PB, Puro Trato, Rio Grande do Sul, Brazil) and filtered water. Before the experimental procedures, mice were acclimatized to the testing room for at least 1 h. Experimental procedures were performed in accordance with ethical guidelines for pain research in conscious animals [44] and followed the Animal Research: Reporting of In Vivo Experiments (ARRIVE) guidelines [45]. The experimental protocol was approved by the Institutional Animal Care and Use Committee of the Federal University of Santa Maria (UFSM, Brazil; protocol no. 2381301120) and complied with the guidelines of the Brazilian National Council for the Control of Animal Experimentation (CONCEA). The animals were numbered from 1 to 40 and randomly assigned using a computer-based random-order generator (www.randomization.com) to four experimental groups: Control-Veh, Control-A-967079, RR-EAE-Veh, and RR-EAE-A-967079, with 10 animals per group. Researchers conducting behavioral assessments and laboratory analyses were blinded to treatment allocation and RR-EAE induction status during these procedures. Behavioral assessments were performed using all animals in the following order: body weight, von Frey test, acetone test, and rotarod test (*n* = 10 per group). At the end of the experimental protocol, subsets of animals were randomly selected for RT-qPCR analyses (*n* = 3 animals per group). In contrast, biochemical and cytokine analyses were performed using *n* = 5 animals per group. No animals were excluded from the study.

### 4.2. Reagents

EZBiolab (Carmel, IN, USA) synthesized Myelin Oligodendrocyte Glycoprotein (MOG35-55) with a chemical purity of 95.57%, according to the manufacturer’s datasheet. Baxter (São Paulo, SP, Brazil) supplied isoflurane, while Syntecvet ( São Paulo, SP, Brazil) supplied xylazine and ketamine, whichwere used for anesthesia and euthanasia. Síntese Biotecnologia (Ribeirão Preto, SP, Brazil) supplied the RT-qPCR primers. All other reagents, unless specified, were obtained from Sigma-Aldrich (St. Louis, MO, USA).

### 4.3. RR-EAE Induction

**Figure 5:**
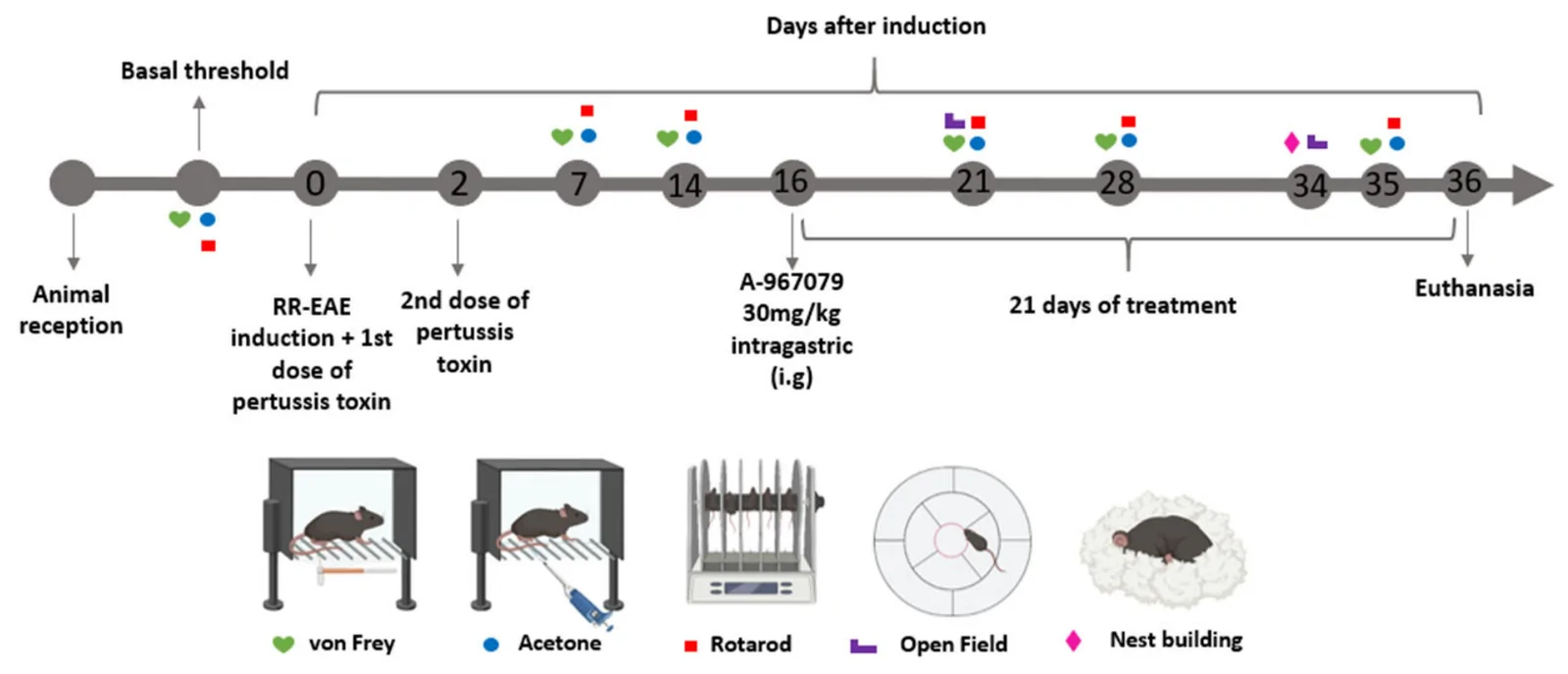
Schematic representation of the experimental procedures, treatment schedule, and behavioral assessments. Created by BioRender. https://BioRender.com/22292fm (accessed on 1 October 2026).

To induce the RR-EAE model, mice were immunized subcutaneously (s.c.) with a mixture of 200 µg of MOG_35-55_ antigen and 45 µg of the adjuvant Quillaja saponin (Quil A) solution diluted in 100 µL of phosphate-buffered saline (PBS) [21]. The mixture was split into two 50 µL aliquots and administered into each flank on day 0 (first day of immunization). On day 0, mice also received 250 ng of pertussis toxin (25 ng/μL in PBS) via intraperitoneal (i.p.) injection and again on day 2 (48 h post-induction). Animals in the control group received the corresponding doses of Quil A (without MOG_35–55_ antigen) and pertussis toxin [21]. RR-EAE-induced groups were treated via intragastric (i.g.) administration with either A-967079 or its vehicle (1% DMSO in 0.9% isotonic saline). After RR-EAE induction, we monitored animals weekly for changes in body weight. We established a 20–30% reduction in body weight from baseline as an exclusion criterion, as previously described [21,22,28]. However, we excluded no animals. The experimental design illustrates the study protocol (Figure 5).

### 4.4. Treatment Protocol

A-967079 is a selective TRPA1 antagonist with IC_50_ values of 67 nM and 289 nM at human and rat TRPA1, respectively, and has been reported to exhibit good CNS penetration [46]. Mice received a daily dose of A-967079 (30 mg/kg) via the intragastric (i.g.) route for 21 days (from the 16th to the 36th day of the experimental protocol) between 12:00 and 1:00 p.m. We selected the A-967079 dose used in this study based on previous reports evaluating the efficacy of TRPA1 antagonism in models of pain and inflammation. Studies such as Dalenogare et al. [21], Trevisan et al. [35] and Antoniazzi et al. [33] demonstrated acute antinociceptive effects at 100 mg/kg in different experimental models. In contrast, Zhu et al. [47] reported significant therapeutic effects using a lower dose (30 mg/kg) in a distinct disease model. Based on this evidence, we selected 30 mg/kg to balance efficacy and safety, allow repeated systemic administration, and support the investigation of long-term therapeutic effects in the chronic neuroinflammatory context of RR-EAE.

The solution of A-967079 or its vehicle was prepared fresh daily prior to administration. Animals were randomly assigned to four groups (*n* = 10 per group): Control-Veh, Control-A-967079, RR-EAE-Veh, and RR-EAE-A-967079. Control groups received an equivalent dose of vehicle (1% DMSO in 0.9% isotonic saline). Treatments were administered after the end of all behavioral tests to avoid the acute effects of A-967079. The schematic representation of the experimental procedures, treatment schedule, and behavioral assessments is shown in Figure 5.

### 4.5. Behavioral Tests

Mice behavior assessments were assessed at baseline (day 0) and on days 7,14, 21, 28, 34, and 35, as previous studies indicate that nociception and neuroinflammation occur during this period [13,20,21]. Animals were acclimated to the experimental room for at least 1 h before testing, and baseline (day 0) measurements were obtained for specific behavioral assessments to enable comparisons over time. All behavioral tests were conducted between 8:00 a.m. and 12:00 p.m. before the next A-967079 treatment administration. The nest-building test was conducted during the dark phase of the light–dark cycle, from 7:00 p.m. to 7:00 a.m., to ensure consistent experimental conditions and alignment with the natural active period of the animals.

#### 4.5.1. Rotarod Test

To assess the forced locomotor activity and balance of the animals, the rotating cylinder test (Rotarod) was performed on days 0 (baseline), 7, 14, 21, 28, and 35 [21]. Animals were trained on the rotarod apparatus one day before RR-EAE induction. Training consisted of placing the animal in the rotating cylinder for 60 s, at a fixed speed of 16 rpm, during which the latency to fall was recorded. On testing days, mice were placed in the rotating cylinder at a fixed speed of 16 rpm for 180 s, and their latency to fall was recorded. Animals that were unable to remain for 180 s on the rotarod were excluded from the study [13]. However, no exclusions were necessary, as all animals completed the test duration.

#### 4.5.2. Von Frey Test for Mechanical Allodynia Detection

To analyze sensitivity to mechanical stimuli, the test was conducted at baseline (day 0) and on days 7, 14, 21, 28, and 35 using a series of seven von Frey filaments of increasing intensity (0.07, 0.16, 0.40, 0.60, 1.0, 1.4, and 2.0 g) [21]. Briefly, animals were acclimated for 60 min before testing in elevated individual acrylic chambers with a metal mesh floor. Plantar mechanical allodynia was performed using the “*up and down*” method on the hind paw of each animal. Testing began with a 0.6 g filament; if the paw was withdrawn, a lower-pressure filament was applied. If the paw was not withdrawn, a higher-pressure filament was used. This process continued for six measurements or until four consecutive positive or negative responses occurred. The results were used to calculate each animal’s 50% mechanical threshold (in grams) [48].

#### 4.5.3. Acetone Test for Assessment of Cold Allodynia

The acetone test was used to assess cold thermal allodynia on days 0, 7, 14, 21, 28, and 35 post-induction. Baseline (day 0) measurements were obtained before the RR-EAE induction. Animals were placed in transparent boxes on an elevated wire mesh platform and acclimated for 60 min before testing. The procedure consisted of applying 20 μL of acetone to the plantar surface of the hind paw. The time spent lifting, licking, or shaking the paw was recorded for 60 s [13].

#### 4.5.4. Open Field Test

The open field test was conducted to assess locomotor and exploratory activity on days 21 and 34 after disease induction, as previously described by Rodrigues et al. [13]. Mice were individually placed in the center of the open field apparatus (50 × 50 × 25 cm) and left alone in the room during the test. Animal behavior was recorded for 5 min using a video tracking system and analyzed using the ANY-maze^®^ software (version 7.0) (Stoelting Co., Wood Dale, IL, USA). Locomotor parameters included the total distance traveled and total number of crossings. The software was configured with ethological keyboards to quantify specific exploratory behaviors, such as the number of rearing events (vertical exploration), grooming duration (self-cleaning behavior), and the number of sniffing episodes (rhythmic nose movements directed toward ground exploration), as described by Rodrigues et al. [38]. Between trials, the apparatus was cleaned with a 30% ethanol solution and thoroughly dried to prevent olfactory cues from influencing behavior.

#### 4.5.5. Nest-Building for the Assessment of Spontaneous Nociception and Animal Welfare

On day 34 post-induction, the nest-building test was performed to assess spontaneous nociception and provide a complementary behavioral measure of animal welfare. Nest-building is a spontaneous species-typical behavior associated with thermoregulation, shelter, protection, and reproduction [31]. It can reflect activities of daily living, motivational state, general neurological function, and overall animal well-being. Therefore, nest-building performance was not considered a specific measure of nociception. Unlike the other behavioral assessments, the nest-building test was performed at a single time point, with each mouse being assessed only once during the experimental protocol [13]. This experimental design was adopted to obtain a standardized assessment of nest-building behavior at the end of the behavioral evaluation period. For the test, 2–3 g of compacted cotton fibers was placed in each cage. At 6:00 p.m., mice were transferred to individual test cages, and the lights were turned off from 7:00 p.m. to 7:00 a.m. Nest-building behavior was assessed the following morning (day 35), and nest quality was scored and classified according to the following criteria: 1—cotton piece nearly intact (>90% intact); 2—partially torn cotton pieces (50–90% remains intact); 3—mostly shredded cotton pieces with no identifiable nest area (<50% intact); 4—identifiable nests with walls smaller than 50% of the mouse’s body circumference (>90% torn); and 5—an excellent nest, with more than 90% of the cotton torn and walls higher than 50% of the mouse’s body circumference [13]. Higher nest-building scores indicate better nest-building performance and have been used as a complementary behavioral measure of pain-related alterations and animal welfare [13,31].

### 4.6. Sample Collection

Two hours after the last treatment administration on day 36 post-induction of RR-EAE, C57BL/6 mice were anesthetized with an induction dose of ketamine (100 mg/kg) and xylazine (20 mg/kg) administered i.p. Anesthesia was maintained with 2.5% isoflurane via inhalation. The animals were then euthanized by intracardiac puncture, and blood and spinal cord were collected. Plasma was obtained by centrifuging the blood at 3000 rpm for 10 min [49].

#### 4.6.1. Reverse Transcription-Quantitative Real-Time PCR (RT-qPCR)

RNA extracted from the spinal cord (segments L4-L6) was purified using the standard TRIzol^®^ method. UV absorbance was measured at 260 nm with NanoDrop technology to assess the RNA levels and purity. For reverse transcription, we combined RNA with DNase, DNase buffer, EDTA (Sigma-Aldrich), RNase-free water, 5× iScript mix (containing primers and dNTPs), and 1× iScript enzyme (Bio-Rad, Hercules, CA, USA) for a total volume of 20 µL. The reverse transcription reaction was conducted in a thermocycler (Applied Biosystems) with the following temperature settings: 27 °C for 15 min, 70 °C for 10 min, 25 °C for 5 min, and 42 °C for 30 min. All genes were quantified using GoTaq^®^ Master Mix (Promega Corporation, Madison, WI, USA) on a StepOne™ system (Applied Biosystems, Foster City, CA, USA). Standard thermal cycling parameters were used to amplify each transcript. A standard curve was generated by performing 2-fold serial dilutions of the samples to determine the primers’ efficiency and measure gene expression according to the standard curve results. The samples were analyzed in duplicate, and results were normalized to the average of a reference gene, hypoxanthine-guanine phosphoribosyltransferase (*Hprt*). Relative quantities were calculated using the 2^−ΔΔCT^ method. The specificity of the primers was confirmed through melt curve analysis, which showed single peak performances of PCR products, and by including negative controls which water was used to replace cDNA [50]. Mouse primer set details are listed in Table 2.

#### 4.6.2. Cytokine Level Analysis

Plasma concentrations of IL-2, IL-6, IL-10, TNF-α, IFN-γ, and IL-17 were quantified using the BD CBA Mouse Th1/Th2/Th17 Cytokine Kit (BD Biosciences, San Jose, CA, USA). Samples were acquired on a BD Accuri C6 flow cytometer, and data were analyzed in FlowJo software (version 10.0). A calibration curve was generated from the kit standards to determine cytokine concentrations (pg/mL) [49].

#### 4.6.3. Assessment of NADPH–Cytochrome c Reductase Activity

NADPH–cytochrome c Reductase activity in the spinal cord (segments L4–L6) was determined according to the methodology previously described by [33]. Tissue samples were analyzed using a commercial assay kit (CY0100, Cytochrome C Reductase, NADPH; Sigma-Aldrich, Milan, Italy). Results were expressed as enzymatic activity units per milliliter per milligram of protein (U/mg of protein).

#### 4.6.4. Measurement of TRPA1 Agonists Levels

##### 4-HNE and H_2_O_2_ Dosage

The concentrations of H_2_O_2_ and 4-HNE were quantified in the spinal cord (segments L4–L6). To assess H_2_O_2_ levels, we used the phenol red-horseradish peroxidase (HRPO) method, as described by [51]. Briefly, 25 mM sodium azide was added to the supernatants to inhibit cytochrome c oxidase activity. The homogenates containing sodium azide were then centrifuged at 12,000× *g* for 20 min at 4 °C. Subsequently, a reaction mixture containing the supernatant, 25 μL of phenol red (100 mg/mL), and 5 μL of HRPO (50 mg/mL) was incubated for 10 min at 25 °C in the dark. The reaction was terminated with 1 M NaOH, and absorbance was measured at 610 nm using a SpectraMax i3^®^ microplate reader (Molecular Devices, LLC, San Jose, CA, USA). H_2_O_2_ concentrations were expressed as nanomoles of H_2_O_2_ per milligram of protein, using a standard curve generated from known H_2_O_2_ concentrations. The levels of 4-HNE were determined using the OxiSelect™ HNE Adduct Competitive ELISA Kit (Cell Biolabs, Inc., San Diego, CA, USA). Samples were homogenized according to the manufacturer’s instructions and analyzed by SpectraMax i3^®^ microplate reader (Molecular Devices, LLC.), as described. Results were expressed as the percentage of 4-HNE relative to the control group [21].

##### AOPP Levels Determination

AOPP levels were quantified in plasma and spinal cord samples (segments L4–L6). Before analysis, spinal cord samples (1:4) and plasma (1:10) were diluted in PBS. The assay was performed using the chloramine-T method, measuring absorbance at 340 nm expressed as nmol/L [13].

### 4.7. Statistical Analysis

Data are expressed as the mean ± standard error of the mean (SEM). The Kolmogorov–Smirnov test was used to evaluate data normality for all behaviors and biochemical analysis, and no test for outliers was conducted. For RT-qPCR analysis (*n* = 3 per group) and biochemical analysis (*n* = 5 per group), we could not assess normality due to the small sample size. Mechanical allodynia data were log-transformed to meet parametric assumptions. Parametric data were subjected to one-way ANOVA, followed by Dunnett’s post hoc test (*Iba-1*, *Gfap*, *Olig-1*, H_2_O_2_, 4-HNE, NADPH–cytochrome c reductase activity, AOPP, IL-2, IL-6, IL-17, TNF-α, IFN-γ, IL-10 levels) or a mixed-effects model with the Bonferroni multiple comparisons test (Rotarod, von Frey, acetone, open field parameters). The non-parametric data were analyzed using the Kruskal–Wallis test followed by Dunn’s test as post hoc analyses (nest building test). The sample size was calculated based on previous group studies [10,14], no *p* sample-size or power calculation was performed. Exact *p*-values and 95% confidence intervals are reported alongside the corresponding results.

PCA was performed using standardized variables (mean = 0 and standard deviation = 1) based on the correlation matrix according to Rodrigues et al. [38]. For PCA and correlation analyses, only animals with behavioral and biochemical/molecular matched data were included. Thus, the same five animals per group used in behavioral tests (von Frey, acetone test, nest building behavior, and open field parameters) and biochemical/molecular analyses in spinal cord and plasma samples (AOPP, NADPH–cytochrome c reductase activity, H_2_O_2_, IL-2, IL-6, IL-10, TNF-α, IFN-γ, IL-17 and 4-HNE) (*n* = 5 per group), were used, ensuring that only matched observations from the same individual animals were included in the multivariate analyses. Components with eigenvalues greater than 1 were retained according to the Kaiser criterion. Absolute component loadings greater than 0.5 were used as a descriptive threshold for interpreting each variable’s contribution to the retained components. Given the limited sample size relative to the number of variables, the PCA was considered exploratory, and the resulting patterns were interpreted as hypothesis-generating rather than confirmatory.

For the correlation matrix analysis, Spearman’s correlation coefficients were interpreted based on their magnitude, with a coefficient of 1.0 indicating a perfect correlation and coefficients below 0.8 considered non-robust correlations. Statistical analyses were performed using GraphPad Prism^®^ version 9.0, and *p* < 0.05 was considered statistically significant.

## 5. Conclusions

In the present study, induction of the RR-EAE model reproduced clinically relevant nociceptive behaviors, such as mechanical/cold allodynia and spontaneous nociception, without inducing motor deficits. The animal model also showed pathological features consistent with symptoms observed in RRMS patients, including reduced expression of myelin markers, activation of microglia and astrocytes, and increased inflammation and oxidative stress markers. Repeated administration of the TRPA1 antagonist produced a robust and sustained antinociceptive effect without inducing locomotor impairment or evident adverse effects over the 36-day protocol. This pharmacological profile brings the experimental model closer to a chronic therapeutic scenario, more consistent with the clinical reality of pain management in patients with RRMS. Beyond nociceptive relief, TRPA1 antagonism modulates central components of disease pathophysiology, reducing glial activation, neuroimmune inflammation, and oxidative stress. Taken together, these findings indicate that TRPA1 plays a relevant role in the development and maintenance of neuropathic-like pain associated with the RR-EAE model, reinforcing this channel as a promising therapeutic target for the development of novel pharmacological strategies for MS-associated pain management. Nevertheless, further preclinical studies evaluating different TRPA1 antagonists, multiple dosing regimens, both biological sexes, and complementary experimental models are warranted to validate these findings and support their potential for clinical translation.

## Figures and Tables

**Table 1:** Variable loadings on the first two principal components from the exploratory principal component analysis (PCA) of behavioral, biochemical, oxidative stress, and inflammatory parameters in the RR-EAE model.

Variable	PC1	PC2
VF	−0.970	0.066
Cold allodynia	0.945	−0.132
Nest score	−0.752	0.460
Sniffing (*n*)	−0.843	0.327
Grooming (*n*)	−0.898	0.111
Rearing (*n*)	−0.898	0.095
AOPPs Spinal	0.861	−0.054
AOPPs Plasma	0.720	−0.143
NADPH Spinal	0.836	−0.127
H_2_O_2_ Spinal	0.749	0.324
IL-2 Plasma	0.861	0.432
IL-6 Plasma	0.891	0.259
IL-17 Plasma	0.889	0.310
IFN-γ Plasma	0.756	0.256
TNF-α Plasma	0.927	0.220
IL-10 Plasma	−0.586	0.698
4-HNE Spinal	0.925	0.152
Eigenvalue	12.197	1.480
Variance explained	71.75	8.71

Factor loadings > 0.5 were significant. Two components were retained according to the Kaiser criterion (eigenvalues > 1). Von Frey (VF); advanced oxidation protein products (AOPPs); (H_2_O_2_); Interleukin-2 (IL-2); Interleukin-6 (IL-6); Interleukin-17 (IL-17); interferon-gamma (IFN-γ); tumor necrosis factor-alpha (TNF-α) Interleukin-10 (IL-10); 4-hydroxynonenal (4-HNE); principal component analysis (PCA).

**Table 2:** Forward and reverse primers used on RT-qPCR assays and their respective sequences (5′–3′).

Gene	Sequence Forward (5′–3′)	Sequence Reverse (3′–5′)	Accession Number
*Iba-1*	AGGAGAAAAACAAAGAACACAAGA	CAATCAGGGCAGCTCGGAGATAGC	NM_019467
*Gfap*	TGCAGGAGTACCAGGATCTAC	GATCTGGAGGTTGGAGAAAGTC	NM_010277
*Olig-1*	AAGCGCCATGCAGGACCTGAACT	AGCGTGGCAATCTTGGAGAGCT	NM_016968.4
*Hprt*	GCACACTTTGCTTTCCCTGG	CAACAACAAACTTGTCTGGA	NM_013556

## Data Availability

The datasets generated during and/or analyzed during the current study are available from the corresponding author on reasonable request.

## Abbreviations

4-HNE, 4-hydroxynonenal; AOPPs, advanced oxidation protein products; ARRIVE, Animal Research: Reporting of In Vivo Experiments; CNS, central nervous system; CONCEA, National Council for the Control of Animal Experimentation; EAE, experimental autoimmune encephalomyelitis; H2O2, hydrogen peroxide; HRPO, phenol red–horseradish peroxidase; IFN-γ, interferon gamma; IL-10, interleukin-10; IL-17, interleukin-17; IL-2, interleukin-2; IL-6, interleukin-6; i.g, intragastric; i.p, intraperitoneal; MOG35–55, Myelin–Oligodendrocyte Glycoprotein; MS, multiple sclerosis; PCA, principal component analysis; PBS, phosphate-buffered saline; Quil A, Quillaja saponin; RR-EAE, relapsing–remitting experimental autoimmune encephalomyelitis; RRMS, relapsing–remitting multiple sclerosis; RT-qPCR, reverse transcription–quantitative real-time PCR; s.c, subcutaneous; TNF-α, tumor necrosis factor alpha; TRPA1, transient receptor potential ankyrin 1.
